# Leiomyoma of the fallopian tube found during laparoscopic myomectomy: A case report and review of the literature

**DOI:** 10.3389/fsurg.2023.997338

**Published:** 2023-03-31

**Authors:** Bing Cheng, Rui Wang, Yaoqun Fu, Xi Fu

**Affiliations:** Department of Obstetrics and Gynecology, Center for Reproductive Medicine/Department of Fetal Medicine and Prenatal Diagnosis/BioResource Research Center, Guangdong Provincial Key Laboratory of Major Obstetric Diseases, The Third Affiliated Hospital of Guangzhou Medical University, Guangzhou, China

**Keywords:** fallopian tube neoplasms, fertility, leiomyoma, benign tumors, laparoscopic myomectomy

## Abstract

Leiomyoma of the fallopian tube is an extremely rare benign tumor of the fallopian tube. Because of the small number of cases, it is difficult to calculate their incidence. In this case report, we report a case of leiomyoma of the fallopian tube detected during laparoscopic myomectomy in a 31-year-old female with occasional pelvic pain. The patient was diagnosed with uterine leiomyoma based on a transvaginal ultrasound scan. She was operated and a 3*3 cm mass in the area of the isthmus of the left fallopian tube was observed. Three uterine leiomyomas and one leiomyoma of the fallopian tube were removed. Ultrasound at 6 months postoperatively showed no abnormality. Hysterosalpingo-contrast-sonography (HyCoSy) at 15 months postoperatively showed bilateral fallopian tubes were unobstructed. For those patients with fertility requirements, some fertility-preserving techniques can be used to allow complete resection of the leiomyoma and avoid tubal damage.

## Introduction

Leiomyoma of the fallopian tube, a benign tumor, is extremely rare in contrast to other gynecologic tumors (1). Most leiomyoma of the fallopian tube are asymptomatic, however, some of them may experience abdominal pain or ectopic pregnancy (2, 3). They are usually discovered accidentally during diagnostic laparoscopy or unrelated surgical procedures (2, 4, 5). However, there are few images of it.

## Case report

**Figure 1:**
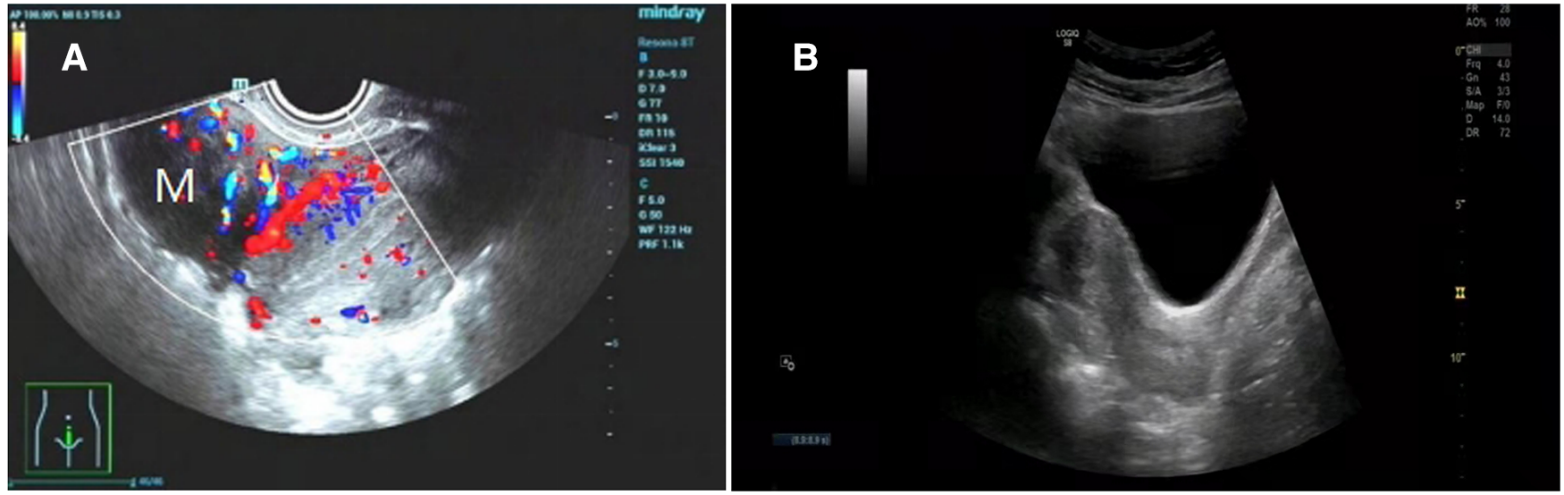
(**A**) Preoperative ultrasound showed multiple uterine leiomyomas. (**B**) Ultrasound image of normal uterus 6 months after surgery.

A 31-year-old Chinese woman, gravida 0, para 0, diagnosed with uterine leiomyoma was admitted to our hospital for surgery. She discovered a 1*1 cm uterine leiomyoma during a physical examination 5 years ago. During her follow-up, the leiomyomas gradually increased in size and increased in number. She had occasional pelvic pain, without previous surgery. Menstruation is normal. A transvaginal ultrasound scan showed multiple uterine leiomyomas, the largest was about 62*42 mm (Figure 1A).

**Figure 2:**
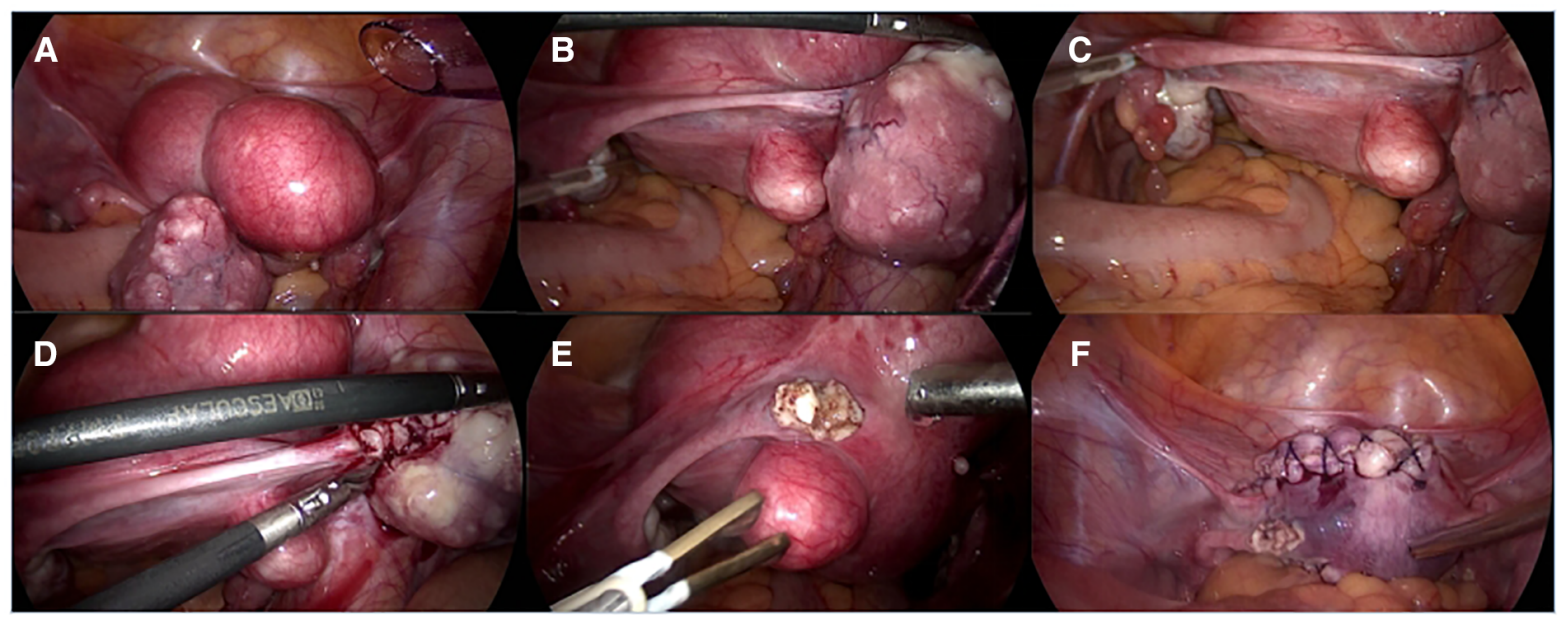
(**A–C**) A 3*3 cm smooth, firm mass with a narrow base was seen in the area of the isthmus of the left fallopian tube. There are also several large uterine leiomyoma, as described by ultrasound. (**D,E**) After the base of leiomyoma of fallopian tube was treated with bipolar coagulation to reduce bleeding, the base was cut 0.5 cm away from the tube by scissors. (**F**) Treat the remaining uterine leiomyoma according to conventional methods.

**Figure 3:**
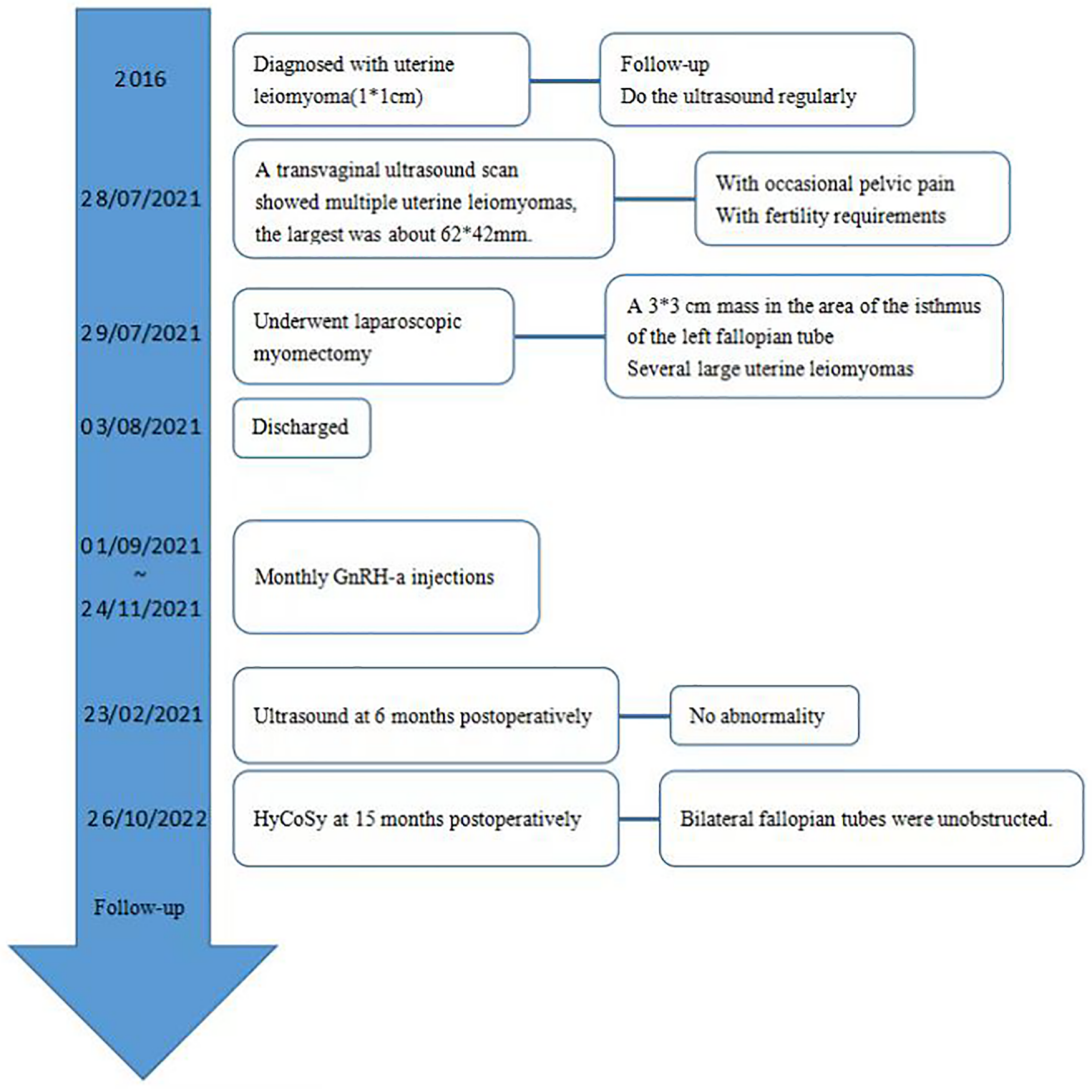
Hysterosalpingo-contrast-sonography (HyCoSy) at 15 months postoperatively showed bilateral fallopian tubes were unobstructed.

She underwent laparoscopic myomectomy. After entering the abdominal cavity, we found a 3*3 cm smooth, firm mass with a narrow base in the area of the isthmus of the left fallopian tube (Figures 2A–C). There are also several large uterine leiomyomas, as described by ultrasound. The base of the leiomyoma of fallopian tube was treated with bipolar coagulation to reduce bleeding, and the base was cut 0.5 cm away from the tube by scissors (Figures 2D,E). This combination of cold and heat ensured the integrity and function of the fallopian tube. The remaining uterine leiomyomas were treat according to conventional methods (Figure 2F). Three uterine leiomyomas and one leiomyoma of the fallopian tube were removed. The final pathology revealed leiomyoma. The patient recovered smoothly and was discharged soon. She received monthly GnRH-a injections for four months after surgery. Ultrasound at 6 months postoperatively showed no abnormality (Figure 1B). Hysterosalpingo-contrast-sonography (HyCoSy) at 15 months postoperatively showed bilateral fallopian tubes were unobstructed (Figure 3).

## Discussion

Benign tumors originating from the fallopian tubes are very rare and can present in a variety of histologic types, including leiomyoma, teratoma, fibroma, Adenomatoid tumor, lipoma, hemangioma, papilloma, Solitary fibrous tumor, etc (6–9). Due to the very small number of cases, it is difficult to assess the incidence of leiomyoma of the fallopian tube. Cases have been reported in the age of onset from reproductive age to menopause, and mostly in 25–45 years old. The highest age reported in the past was 70 years old, reported by Ozkan et al. (10). Leiomyoma of the fallopian tube originates from the smooth muscle of the tube or the cells of the blood vessels supplying the fallopian tube (11). Embryologically, both the uterus and the fallopian tube originate from the Müllerian duct. Part of the reason for the difference in incidence between tubal and uterine leiomyoma may be that the myometrium of the fallopian tube is relatively unresponsive to estrogen compared to the uterine myometrium (2). Leiomyoma of the fallopian tube are mostly unilateral, small and asymptomatic, and most cases are discovered incidentally during diagnostic laparoscopy or other operations. Some patients may seek medical attention due to the huge size of tubal leiomyoma or abdominal pain caused by torsion or degenerative changes of leiomyoma, or due to the coexistence of tubal ectopic pregnancy and tubal leiomyoma (2, 9, 12). The pathogenesis of ectopic pregnancy may be that tubal leiomyoma compresses the lumen of the fallopian tube or tubal leiomyoma lead to alteration in the ciliary motion in the tube so that the fertilized egg implants in the tube. Ozkan et al. reported a case of massive intraperitoneal hemorrhage due to spontaneous rupture of leiomyoma of the fallopian tube (10). Imaging findings of tube leiomyoma were similar to those of uterine leiomyoma. Ultrasound shows a homogeneous hypoechoic solid mass with poor sound through transmission (13). CT showed a sausage-shaped mass separated from the ovary (14). Viable leiomyomas enhance homogeneously. Because the blood supply of the fallopian tube is not as rich as the uterine myometrium, when the growth of the tube leiomyoma exceeds its blood supply, degeneration such as myxoid degeneration often occurs. Degeneration is indicated when coarse calcification or heterogeneous enhancement is present. MR imaging is the best way to visualize and localize leiomyomas. The images show a fusiform adnexal mass between, but separate from, the uterus and ovary. On T1-weighted images, leiomyomas are isointense relative to the uterine myometrium and hypointense on T2-weighted images. With homogeneous enhancement in viable leiomyomas and heterogeneous enhancement in degenerating leiomyomas, and fallopian tube torsion appears as tubular lesions, tapered ends, beak sign (9, 14). Nevertheless, leiomyoma of the fallopian tube is still hard to diagnose preoperatively and is often misdiagnosed as subserous uterine leiomyoma with torsion, ovarian cyst, mesenteric tumor, small pelvic tumor, abdominal hemorrhage, etc. This is partly because primary tumors of the fallopian tube are uncommon, easily diagnosed as a mass in the ovary or uterus, and often require surgical exploration to confirm the diagnosis. When leiomyoma of the fallopian tube is present, the differential diagnosis of this disease should include parasitic leiomyoma and leiomyomatosis peritonealis disseminata (LPD) (15, 16). Patients with parasitic leiomyoma had a history of laparoscopic surgery with the use of morcellation. Parasitic myomas were found on the peritoneum of theabdominal or pelvic wall, in Douglas' pouch or on the omentum, colon and small intestines (16). If patients without a history of laparoscopic morcellation, the differential diagnosis of LPD should be considered. LPD is a disease that is characterised by the presence of many (sub-) peritoneal smooth-muscle nodules disseminated through the omentum and peritoneum (15). The pathogenesis of this disease is still unclear, but the nodules are thought to originate from sub-mesothelial multipotential cells located in the female pelvic peritoneum. In most cases, the leiomyoma is located in the isthmus, a few are located in the ampulla, and most are located in the left fallopian tube (1, 2, 5), as in this case. The surgical method and scope are based on the patient's age, lesion size, and fertility status. Salpingectomy or myomectomy can be selected. Patient with no fertility requirement should consider salpingectomy. When the patient is complicated with uterine disease, total hysterectomy and bilateral salpingectomy or bilateral adnexectomy can be considered. Most tubal leiomyoma can be removed by laparoscopy, unless patients have contraindications to laparoscopic surgery, such as severe pelvic adhesions. It should be suspected that the lesion is malignant if the intraoperative leiomyoma is fish-like, with unclear boundaries, brittle quality, and abundant blood supply. The surgical scope should be determined according to the frozen section diagnosis. Especially for patients with fertility requirements, the leiomyoma should be removed as much as possible on the premise of ensuring the integrity and function of the fallopian tube. Before the surgery, the use of vasopressin can decrease bleed loss during surgery. The use of chromopertubation can define the tube and avoid the lumen, allowing complete resection of the leiomyoma, and the avoidance of electrosurgery around the tubes. Cellular leiomyoma is a morphologic variant of tubal leiomyoma reported in the literature and characterized by a dense cellular infiltrate composed of spindle or round cells with scant cytoplasm, nuclei without atypia, low mitotic activity, and a less obvious interlacing fascicle pattern (5, 10). No adjuvant therapy is required after surgery, but long-term follow-up is required due to its malignant potential (17).

## Data Availability

The original contributions presented in the study are included in the article/Supplementary Material, further inquiries can be directed to the corresponding author/s.

## References

[1] Honoré LH Dunnett IP. Leiomyoma of the fallopian tube. A case report and review of the literature. Arch Gynakol. (1976) 221(1):47–50. 10.1007/BF00667680 989266

[2] Misao R Niwa K Iwagaki S Shimokawa K Tamaya T. Leiomyoma of the fallopian tube. Gynecol Obstet Invest. (2000) 49(4):279–80. 10.1159/000010261 10828715

[3] Schust D Stovall DW. Leiomyomas of the fallopian tube. A case report. J Reprod Med. (1993) 38(9):741–2. 10.1186/1752-1947-4-181 8254601

[4] Wen KC Yang CC Wang PH. Primary fallopian tube leiomyoma managed by laparoscopy. J Minim Invasive Gynecol. (2005) 12(3):193. 10.1016/j.jmig.2005.03.020 15922972

[5] Sikora-Szczęśniak DL. Leiomyoma and leiomyoma cellulare of the fallopian tube: review of the literature and case reports. Prz Menopauzalny. (2016) 15(3):143–7. 10.5114/pm.2016.63053 27980525 PMC5137476

[6] Green TH Jr Scully RE. Tumors of the fallopian tube. Clin Obstet Gynecol. (1962) 5:886–906. 10.1097/00003081-196209000-00022 13901452

[7] Youngs LA Taylor HB. Adenomatoid tumors of the uterus and fallopian tube. Am J Clin Pathol. (1967) 48(6):537–45. 10.1093/ajcp/48.6.537 6074219

[8] Berzal-Cantalejo F Montesinos-Carbonell M Montesinos-Carbonell ML Calabuig-Crespo C Martorell-Cebollada MA. Solitary fibrous tumor arising in the fallopian tube. Gynecol Oncol. (2005) 96(3):880–2. 10.1016/j.ygyno.2004.11.020 15721444

[9] Kwon GH Rha SE Ki EY Bae SN Lee A. Imaging findings of fallopian tube leiomyoma with myxoid degeneration: a case report. Clin Imaging. (2015) 39(6):1119–22. 10.1016/j.clinimag.2015.07.003 26271147

[10] Ozkan Z Gonen AN Emir S Yazar FM Gul E Artas ZD Spontaneous rupture of tubal leiomyoma causing haemoperitoneum. J Coll Physicians Surg Pak. (2014) 24(Suppl 2):S91–2. PMID: 24906284. 24906284

[11] Sikora-Szczęśniak DL. Leiomyoma and leiomyoma cellulare of the fallopian tube: review of the literature and case reports. Prz Menopauzalny. (2016) 15(3):143–7. 10.5114/pm.2016.63053 27980525 PMC5137476

[12] Joshi U Kiwalkar S Joshi R. Coexistence of a tubal ectopic pregnancy and tubal leiomyoma. J Minim Invasive Gynecol. (2019) 26(2):350–1. 10.1016/j.jmig.2018.04.016 29704678

[13] Yang CC Wen KC Chen P Wang PH. Primary leiomyoma of the fallopian tube: preoperative ultrasound findings. J Chin Med Assoc. (2007) 70(2):80–3. 10.1016/S1726-4901(09)70307-7 17339150

[14] Rezvani M Shaaban AM. Fallopian tube disease in the nonpregnant patient. Radiographics. (2011) 31(2):527–48. 10.1148/rg.312105090 21415195

[15] Rosati A Vargiu V Angelico G Zannoni GF Ciccarone F Scambia G Disseminated peritoneal leiomyomatosis and malignant transformation: a case series in a single referral center. Eur J Obstet Gynecol Reprod Biol. (2021) 262:21–7. 10.1016/j.ejogrb.2021.05.006 33989940

[16] Van der Meulen JF Pijnenborg JM Boomsma CM Verberg MF Geomini PM Bongers MY. Parasitic myoma after laparoscopic morcellation: a systematic review of the literature. BJOG. (2016) 123(1):69–75. 10.1111/1471-0528.13541 26234998

[17] Guan R Zheng W Xu M. A retrospective analysis of the clinicopathologic characteristics of uterine cellular leiomyomas in China. Int J Gynaecol Obstet. (2012) 118(1):52–5. 10.1016/j.ijgo.2012.01.023 22520514

